# Comparison of Biochemical Characteristics, Action Models, and Enzymatic Mechanisms of a Novel Exolytic and Two Endolytic Lyases with Mannuronate Preference

**DOI:** 10.3390/md19120706

**Published:** 2021-12-14

**Authors:** Lianghuan Zeng, Junge Li, Yuanyuan Cheng, Dandan Wang, Jingyan Gu, Fuchuan Li, Wenjun Han

**Affiliations:** 1 National Glycoengineering Research Center, Shandong Key Laboratory of Carbohydrate Chemistry and Glycobiology, NMPA Key Laboratory for Quality Research and Evaluation of Carbohydrate-Based Medicine and State Key Laboratory of Microbial Technology, Shandong University, Qingdao 266237, China; zenglianghuan2018@163.com (L.Z.); junge_li2020@163.com (J.L.); wddan2015@163.com (D.W.); fuchuanli@sdu.edu.cn (F.L.); 2 United Post-Graduate Education Base of Shandong University and Jinan Enlighten Biotechnology Co., Ltd., Jinan 250100, China; cyysdu@163.com (Y.C.); gujingyan76@aliyun.com (J.G.); 3 Department of Food Science and Engineering, Shandong Agriculture and Engineering University, Jinan 250100, China; 4 Activity Biotechnology Co., Ltd., Jinan 250100, China

**Keywords:** action mode, catalytic mechanism, gene truncation, oligosaccharide-yielding property, protein-structure modeling

## Abstract

Recent explorations of tool-like alginate lyases have been focused on their oligosaccharide-yielding properties and corresponding mechanisms, whereas most were reported as endo-type with α-L-guluronate (G) preference. Less is known about the β-D-mannuronate (M) preference, whose commercial production and enzyme application is limited. In this study, we elucidated Aly6 of *Flammeovirga* sp. strain MY04 as a novel M-preferred exolytic bifunctional lyase and compared it with AlgLs of *Pseudomonas aeruginosa* (Pae-AlgL) and *Azotobacter vinelandii* (Avi-AlgL), two typical M-specific endolytic lyases. This study demonstrated that the AlgL and heparinase_II_III modules play indispensable roles in determining the characteristics of the recombinant exo-type enzyme rAly6, which is preferred to degrade M-enriched substrates by continuously cleaving various monosaccharide units from the nonreducing end, thus yielding various size-defined ΔG-terminated oligosaccharides as intermediate products. By contrast, the endolytic enzymes Pae-rAlgL and Avi-rAlgL varied their action modes specifically against M-enriched substrates and finally degraded associated substrate chains into various size-defined oligosaccharides with a succession rule, changing from ΔM to ΔG-terminus when the product size increased. Furthermore, site-directed mutations and further protein structure tests indicated that H^195^NHSTW is an active, half-conserved, and essential enzyme motif. This study provided new insights into M-preferring lyases for novel resource discoveries, oligosaccharide preparations, and sequence determinations.

## 1. Introduction

Alginate contributes approximately 40% of seaweed’s dry weight [1]. Alginate is composed of β-(1,4)-linked uronic residues, i.e., β-d-mannuronate (M) and its C5-epimer α-l-guluronate (G), and thus forms poly-M, poly-G, poly-MG, and poly-GM blocks within the linear macromolecule [2,3,4]. Algal alginate, particularly in the form of G-enriched polysaccharides, has been widely applied in the food and pharmaceutical industries due to its excellent gel-forming capability and various associated beneficial effects [5,6,7,8,9]. By contrast, M-enriched alginate oligosaccharides have been identified with important biological activities, e.g., antibacterial [10], anti-obesity [11], antioxidation [12], and anti-inflammatory effects [13], which are closely related to molecular sizes (degrees of polymerization, DPs), M/G ratios, and molecular modification types, e.g., acetylation or sulfation. In 2019, GV-971 [14], a novel drug derived from oligo-mannuronate, was permitted for sale in China to treat Alzheimer’s disease, which makes it of great economic value. Thus, the direct preparation of sugar chains with designated sugar components and molecular sizes from alginate has become an urgent technical problem.

Compared to chemical and physical methods [15,16,17], enzymatic strategies of oligosaccharide preparation have attracted attention for their environmental benefits and less-disordered and preserved sugar units at chain ends [18,19,20]. Alginate lyase can degrade alginate at β-(1,4)-glycoside linkages via a β-elimination mechanism [21]. In general, endolytic alginate lyases produce a series of size-defined oligosaccharide fractions as final alginate digests, with unsaturated units (Δ) at the newly formed nonreducing (*nr*) end; therefore, they are important for the preparation of various unsaturated oligosaccharide chains [22,23,24]. By contrast, throughout the alginate degrading process, exolytic alginate lyases primarily yield the unsaturated monosaccharide product of Δ, which is further converted into 4-deoxy-L-erythro-5-hexoseulose uronate (DEH) under enzymatic conversions [25] and, further, into biofuels [26,27]; thus, exolytic enzymes are essential for the bioconversion of alginate into biofuels to support bacterial growth. Hence, an increasing number of endolytic alginate lyases have been explored as resources and partially improved as tool-like enzymes with respect to their substrate preferences, oligosaccharide-yielding properties, and corresponding substrate-degrading modes [28,29,30,31]. However, relatively few exolytic enzymes are known, which are essential to clearly and exactly display the utility of enzymes in the direct preparation of targeting unsaturated oligo-alginate chains.

For instance, the G-preferring endolytic alginate lyases, Aly5 [29], Aly1 [30], and Aly2 [31], from the same marine-derived polysaccharide-degrading bacterium, *Flammeovirga* sp. strain MY04 [32], are considered valuable for the preparation of large and bioactive unsaturated oligoalginates. Notably, fewer than five endolytic enzymes have been well elucidated for oligosaccharide preparation purposes [29,30,31], although tens of alginate lyases have been reported with G specificity or G preference in recent decades [33,34], which is an even lower proportion than the number of reported lyases with M specificity or M preference. Therefore, we are interested in the utility and associated catalytic mechanisms of M-preferring lyases and, furthermore, differences between endolytic and exolytic enzymes.

A number of bacterial strains of the *Pseudomonas* and *Azotobacter* genera can secrete extracellular alginate, which contains acetyl modification at the O-2 or O-3 sugar ring positions [35,36,37,38] and is an important component in drug-resistant bacterial biofilms. These bacteria encode periplasmic alginate lyases (AlgLs) of the PL5 family through the algL gene, which is localized within alginate biosynthesis operons. In the CAZy databank (http://www.cazy.org/Polysaccharide-Lyases.html, accessed on 1 December 2021), eight AlgL module-containing proteins of the PL5 family have been reported to feature crystal structures, key active site residues (e.g., the half-conserved NNHSYW motif) [39,40,41], and associated functional roles in catalysis. Moreover, an increasing number of AlgL-conserved PL5 proteins have been widely identified through molecular mining of bacterial genomic data. However, relatively little is known about these enzymes’ oligosaccharide products and corresponding substrate action modes, except their M-specificities or M-preferences. This lack of knowledge urgently needs to be overcome in order to achieve direct oligosaccharide preparation, structure identification, and functional exploration by using these AlgLs.

In this study, wild-type, M-preferred and endolytic genes of AlgL from *Pseudomonas aeruginosa* (Pae-AlgL) and *Azotobacter vinelandii* (Avi-AlgL) respectively, sharing the same key motif of NNHSYW at the *C*-terminus, were initially codon-optimized, artificially synthesized, cloned and expressed in *Escherichia coli* strain BL21(DE3) for soluble proteins and comparison to the protein Aly6 of the marine-derived polysaccharide-degrading bacterium *Flammeovirga* sp. strain MY04. The resulting recombinant proteins were individually purified for comparative studies on their biochemical characteristics, enzymatic properties (substrate preference, substrate-degrading modes, and oligosaccharide products), and, particularly, the relationships with their structures. Furthermore, gene truncations and site-directed mutations were performed by homology-based protein-structure modeling and enzyme-substrate stockings to predict enzymatic function changes and tests to determine associated catalytic mechanisms.

## 2. Results

### 2.1. Sequence Characteristics of the Alginate Lyases

**Figure 1:**
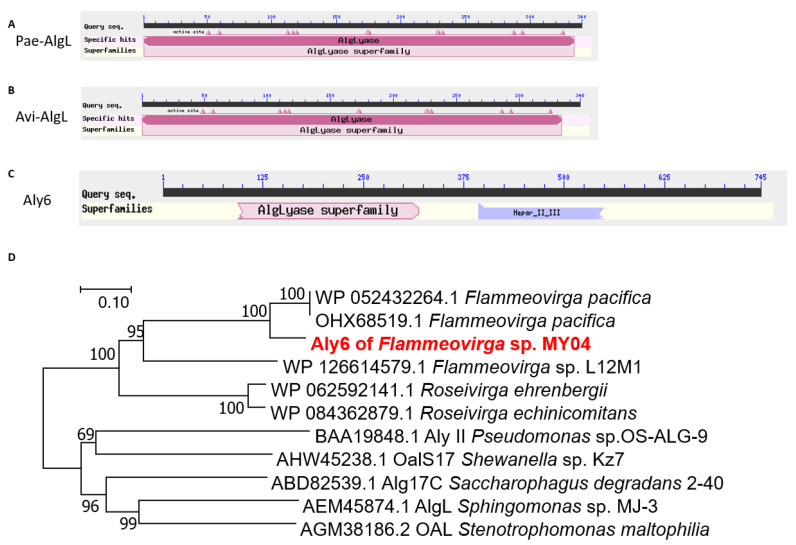
Sequence characteristics of alginate lyases Aly6, Pae-AlgL, and Avi-AlgL. (**A**) Modular organization architecture of Pae-AlgL; (**B**) modular organization of Avi-AlgL; (**C**) modular organization of Aly6; (**D**) phylogenetic analysis of alginate lyases. The phylogenetic tree was constructed using MEGA version 7.2.5 software via the neighbor-joining algorithm, and associated taxa clustered together in a bootstrap test of 1000 replicates.

ORF2549 in the genome of *Flammeovirga* sp. strain MY04 was predicted to encode a candidate polysaccharide lyase, Aly6 (GenBank: ANQ49918.2). The full-length gene was 2238 bp with a GC content of 36.8%. The putative protein Aly6 contained 745 amino acid residues with an apparent molecular mass of 84.67 kDa. The predicted isoelectric point (pI) value was 7.3. SignalP 5.0 analysis indicated that the signal peptide of Aly6 was composed of 23 amino acid residues (Met^1^ to Ser^23^) (Figure 1C).

Aly6 contained a putative *N*-terminal catalytic module (Ala^76^ to Phe^320^) associated with PL5 alginate lyases (AlgL), as well as a putative *C*-terminal module (Leu^391^ to Val^664^) associated with heparinase II or III (Hep II_III) from *Pedobacter heparinus* (Figure 1C). BLASTp searches showed that among the characterized enzymes, Aly6 shared the greatest sequence identity (38.93%) with the exo-type alginate lyase Alg17c (GenBank: ABD82539.1) of *Saccharophagus degradans* strain 2–40, followed by 38% down to 35% identities, with four reported PL17 family alginate lyases (Figure 1D): OalS17 (GenBank: AHW45238.1) of *Shewanella* sp. strain Kz7, AlgL (GenBank: AEM45874.1) of *Sphingomonas* sp. strain MJ-3, OAL (GenBank: AGM38186.2) of *Stenotrophomonas maltophilia*, and AlyII (GenBank: BAA19848.1) of *Pseudomonas* sp. strain OS-ALG-9. Subsequently, Aly6 shared very low sequence identities with PL15 and PL5 family alginate lyases (Table S2). Interestingly, the PL17 proteins described above were all organized in the same (an AlgL and Hep_II_III) complex modular architecture, similar to that of Aly6. Notably, Aly6 shared no homology with the studied PL6 or PL7 alginate lyases, including Aly1 [30], Aly2 [31], and Aly5 [29], from the same *Flammeovirga* sp. strain MY04, which contained neither AlgL-like nor Hep_II_III-like modules.

Furthermore, protein sequence alignment showed that the AlgL module of Aly6 contained one putative catalytic motif, H^195^N^196^H^197^S^198^T^199^W^200^, which was half-conserved but different from the conserved NNHSYW catalytic motif at the C-terminus of diverse PL5 alginate lyases [39,40,41]. Additionally, the potential HNHG(A)TW catalytic motif was half-conserved among various PL17 alginate lyases (Figure S1). Phylogenetic analyses indicated that Aly6, together with six other genome-predicted alginate lyases that were organized in the same AlgL and Hep_II_III complex modular architecture, i.e., four proteins of other *Flammeovirga* strains, one of *Roseivirga ehrenbergii* and the other of *R. echinicomitans*, were clustered into the same novel separate branch within the PL17 superfamily (Figure 1D).

### 2.2. Production and Purification of the Full-Length and Truncated Recombinant Proteins

SDS–PAGE analyses indicated that BL21(DE3) cells harboring each of the above recombinant plasmids produced soluble proteins (Figure S2), with the correct apparent molecular mass and yields greater than 1.0 g/L. After sonication and centrifugation, crude enzymes were extracted from the *E. coli* cultures. Soluble protein fractions containing rAly6, rTF-Aly6, rTF-Aly6-Lmodule, rTF-Aly6-HPmodule, Pae-rAlgL, and Avi-rAlgL were individually eluted from a Ni-nitrilotriacetic acid (NTA) column using imidazole at concentrations above 50 mM. Further SDS–PAGE analyses indicated that the purified soluble proteins each featured purities greater than 95% (Figure S2).

### 2.3. Enzyme Characteristics of the Recombinant Proteins

The recombinant proteins rAly6 and rTF-Aly6 showed the same substrate spectrum, in which they did not digest any tested glycosaminoglycans, i.e., chondroitin sulfates (A, C, D, and E types), dermatan sulfate B, hyaluronan, heparin, heparin sulfate (data not shown), but efficiently digested alginate and the poly-M block, as well as a few of the poly-G blocks to produce unsaturated oligosaccharide products, exhibiting strong absorbance at 235 nm. These results suggested that the protein Aly6 of *Flammeovirga* sp. strain MY04 is an M-preferred bifunctional alginate lyase. Furthermore, the enzyme activity tests on rAly6 and rTF-Aly6 indicated a similar substrate preference for M over G (Table 1). Unlike the whole proteins, i.e., the rAly6 and rTF-Aly6, the gene-truncated proteins, i.e., the rTF-Aly6-Lmodule and rTF-Aly6-HPmodule, did not show any degradation activity against any tested polysaccharides (Table 1), indicating that both the AlgL-like module and the Hep_II_III-like module are indispensable for the alginate lyase activity of Aly6. Furthermore, these results demonstrated that in Aly6, the Hep_II_III-like module is only a putative element instead of a catalytic module against any tested glycosaminoglycans. Recombinant endo-type enzymes, i.e., Pae-rAlgL and Avi-rAlgL, could efficiently degrade alginate and only digest poly-M blocks to yield unsaturated oligosaccharide products (Table 1), demonstrating that they are M-specific lyases.

The recombinant enzyme rAly6 demonstrated the highest activity at 40 °C when alginate was used as the substrate (Figure S3A). A thermostability assay further showed that the alginate-degrading activity of rAly6 was stable at 0 °C to 30 °C, and more than 60% activity was retained even if the enzyme was incubated at 30 °C for 24 h (Figure S3C). The optimal pH, determined at 40 °C in 50 mM buffers of NaAc-HAc and NaH_2_PO_4_-Na_2_HPO_4_, was 6.0 (Figure S3B). The enzyme retained more than 60% of its highest activity after preincubation for 2 h at pH 5.0 to 8.0 (Figure S3B). We also found that the TF-factor-fused recombinant enzyme rTF-Aly6 showed similar biochemical characteristics, i.e., the optimal temperature and pH value for catalysis and the enzyme’s thermal and pH stabilities, to rAly6 (data not shown).

The activity of rAly6 was strongly inhibited by 1 mM or 10 mM Ag^+^, Cu^2+^, Hg^2+^, sodium dodecyl sulfonate (SDS), and 10 mM Pb^2+^, Zn^2+^, Cr^3+^, Fe^3+^, or ethylenediaminetetraacetic acid (EDTA). By contrast, the enzyme activities of rAly6 were increased to 130~160% by various concentrations (1.0 or 10 mM) of Co^2+^, Mn^2+^, and Ni^2+^. Chemicals such as glycerol, dithiothreitol (DTT), and the reducing agent β-mercaptoethanol (β-ME) weakly increased the activity of rAly6 (Figure S3E). Moreover, the activity of rAly6 was strongly increased by NaCl concentrations from 0.0 M to 0.5 M; however, it was inhibited from 0.5 M to 1.0 M (Figure S3D). The enzyme activities of Avi-rAlgL and Pae-rAlgL were both strongly inhibited by 1.0 mM, 10 mM SDS, or 10 mM Cu^2+^, Hg^2+^, Pb^2+^, Zn^2+^, Cr^3+^, and Fe^3+^ (Figure S4A,B) and increased by NaCl concentrations from 0 M to 1.0 M (Figure S4C,D).

Under optimal conditions (40 °C in 50 mM NaAc-HAc buffer, pH 6.0), the enzyme rAly6 showed specific activities of ~726 ± 2.2, ~525 ± 3.5, and ~196 ± 2.9 U/mg in the degradation of alginate, the poly-M block, and the poly-G block, individually. These results demonstrated that the protein Aly6 is an M-preferring lyase. Similarly, the reported M-specific lyases Pae-rAlgL and Avi-rAlgL exhibited activities of ~2685 ± 3.6 and 4219 ± 1.8, ~5704 ± 4.5 and 8085 ± 5.9, and ~144 ± 3.3 and 98 ± 5.2 U/mg in the individual degradation of the corresponding substrates (Table 1).

### 2.4. Degradation Patterns

In alginate degraded by the recombinant enzymes Avi-rAlgL and Pae-rAlgL, unsaturated oligosaccharides with high DPs were the main products in the initial steps and gradually converted into smaller products (Figure S5A,B), eventually producing various size-defined fractions (Figure S5A,B,D), indicating that Avi-AlgL and Pae-AlgL are endo-type alginate lyases, which is in agreement with existing reports [39,40,42,43]. By contrast, rAly6 exhibited quite different degradation behavior (Figure S5C), suggesting that the Aly6 protein is an exo-type alginate lyase.

**Figure 2:**
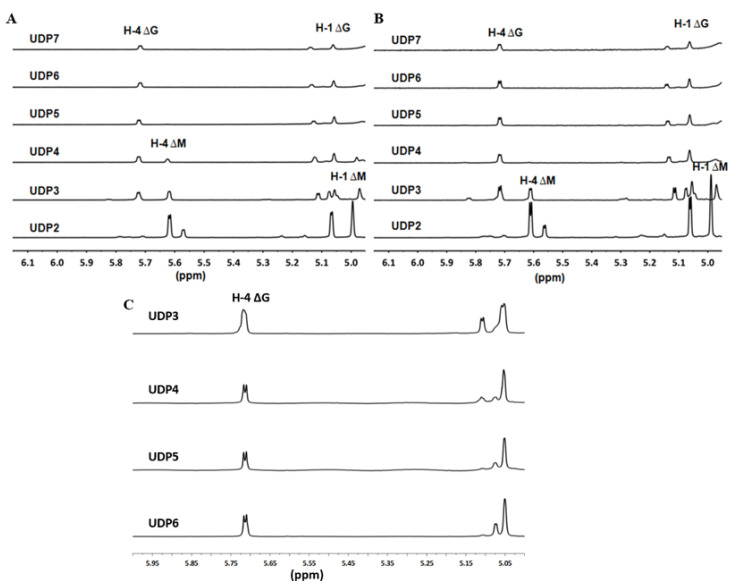
^1^H-NMR analyses of oligosaccharide products. (**A**) ^1^H-NMR (600 MHz) spectra of the final main final products of UDP2~UDP7 fractions individually purified from alginate digests by Pae-rAlgL. (**B**) Final main products of UDP2~UDP7 fractions by Avi-rAlgL. (**C**) Intermediate products of UDP3~UDP6 fractions by rAly6.

The ^1^H-NMR chemical shifts of the proton within the ∆ units at the *nr* end of the unsaturated oligo-alginate chains were strongly affected by the properties of the nearest monosaccharide residues and the residual structures next to each ∆ unit [30,44,45,46]. In addition, chemical shifts at 5.62 ppm indicated that UDP2 (unsaturated disaccharide; U, unsaturated) fractions, small size-defined final products produced by endolytic enzymes Avi-rAlgL or Pae-rAlgL, were ΔM units. The 5.70 ppm signals of UDP5~UDP7 fractions, large size-defined final products by Pae-rAlgL, or of UDP4~UDP7 fractions, large size-defined final products by Avi-rAlgL, indicated that they were ΔG-ended (Figure 2A,B). Furthermore, UDP3’s final products by Avi-rAlgL were determined to contain both ΔM- and ΔG-ends, with a molar ratio of 134:100 (Figure 2B). Additionally, UDP3’s and UDP4 final products by Pae-rAlgL contained these two different end types, with ratios of 100:104 and 100:196, respectively (Figure 2A). However, the larger size-defined final unsaturated oligo-alginate products of these two endolytic enzymes contained only ΔG-ends (Figure 2A,B). In the case of the purified UDP3~UDP6 fractions, the intermediate products by rAly6, specific signals at 5.65 ppm of H-4 of ∆G were strong (Figure 2C), but no ∆M signals were found, demonstrating that Aly6 is a novel alginate lyase that produces a series of ∆G-terminal oligosaccharides as intermediate products. Therefore, the novel exolytic enzyme Aly6 is valuable for yielding ΔG-ended intermediate oligosaccharide products, while the endolytic enzymes, Pae-AlgL and Avi-AlgL, helpf to yield final oligosaccharide products with succession rules, changing from ΔM- to ΔG-ends when the product size increases.

**Figure 3:**
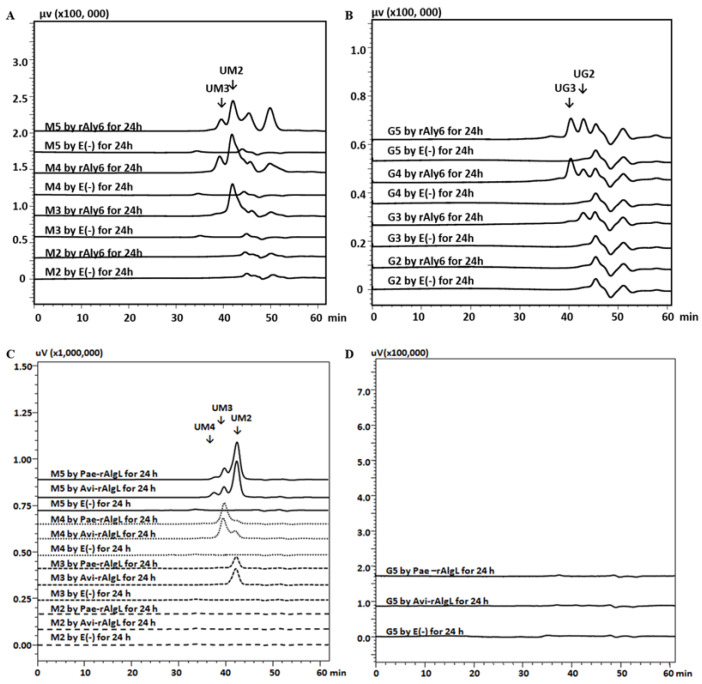
Substrate preferences of rAly6, Pae-rAlgL, and Avi-rAlgL. (**A**) Saturated M2~M5 substrate chains reacted with rAly6; (**B**) saturated G2~G5 chains reacted with rAly6; (**C**) saturated M2~M5 reacted with Pae-rAlgL and Avi-rAlgL; (**D**) saturated G5 reacted with Pae-rAlgL and Avi-rAlgL. E (−), control treated with inactivated enzymes.

To further investigate the different substrate action models of these three alginate lyases, various size-defined saturated oligosaccharides (M2~M5, G2~G5) were individually used as testing substrates. As a result, the endolytic enzymes Pae-rAlgL and Avi-rAlgL could digest M-enriched oligosaccharides (M3-M5) but did not degrade any tested G-enriched substrate chains (Figure 3C,D), demonstrating that Pae-AlgL and Avi-AlgL are indeed M-specific alginate lyases [39,40,42,43]. They could not digest M2 but could degrade the minimal saturated oligosaccharide substrate M3 and, therefore, yield the smallest saturated product of monosaccharide M. According to our HPLC analyses and further peak area integrals, the main product of the M3 substrate was determined to be a UM2 chain produced from the r end. The same UM3 product was produced from the M4 substrate (Figure 3C). When the M5 chains were degraded using Pae-rAlgL or Avi-rAlgL, the final products were mainly UM2 chains, with a small amount of UM3 and UM4 fractions as products. By contrast, the enzyme rAly6 could degrade both saturated M-enriched oligosaccharides (larger than disaccharides) but could digest a small proportion of the tested G-enriched oligosaccharide fractions (Figure 3A,B), almost to the former, at an enzyme activity of ~5–10:1 at equal molar concentrations, demonstrating that Aly6 is an M-preferred bifunctional exolytic alginate lyase. Accordingly, the oligosaccharide products, including the largest saturated and the final main unsaturated product, enlarged their sizes with increasing substrate size (Figure 3A), demonstrating that Aly6 is an enzyme with a variable action model [30,31].

**Figure 4:**
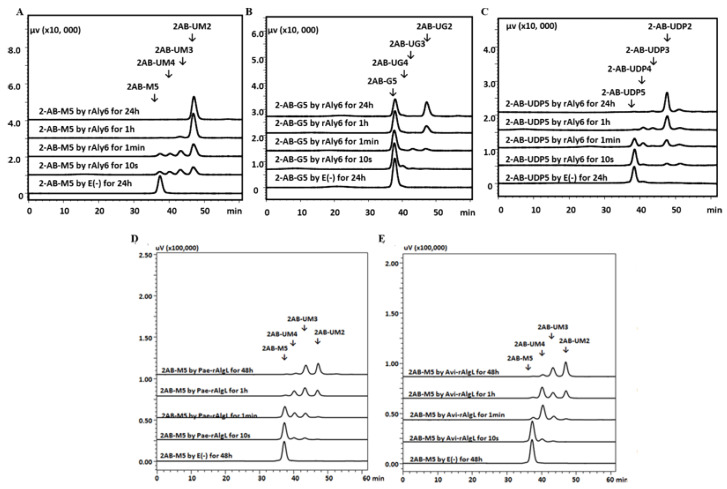
Fluorescent HPLC analyses of the degradation orientation of rAly6, Pae-rAlgL, and Avi-rAlgL. (**A**) 2-AB-M5 degraded by rAly6; (**B**) 2-AB-G5 degraded by rAly6; (**C**) 2-AB-UDP5 degraded by rAly6; (**D**) 2-AB-M5 degraded by Pae-rAlgL; (**E**), 2-A B-G5 degraded by Avi-rAlgL. E (−), control treated with inactivated enzymes accordingly. The resulting products were analyzed on a Superdex^TM^ peptide 30 Increase 10/300 GL gel filtration column monitored using a fluorescent detector with an excitation wavelength of 330 nm and an emission wavelength of 420 nm.

The gel filtration HPLC assays showed that the digestion of 2-AB-M5 and 2-AB-UDP5 substrates by rAly6 yielded a series of 2-AB-labeled oligosaccharide products with high DPs at the beginning, subsequently, and until smaller product chains of 2-AB-UM2 and 2-AB-UDP2 were produced (Figure 4A,C). Therefore, the molar proportion of the 2-AB-UM2 and 2-AB-UDP2 products was produced initially and gradually increased along with the reaction time. For the digestion of 2-AB-G5 by rAly6, ~45% of the substrate chains were finally degraded into 2-AB-UG2 products in equimolar amounts (Figure 4B). The results demonstrated that Aly6 cleaves the 2-AB-M5 or the 2-AB-UDP5 substrate chains using a monosaccharide-yielding exo-type; that is, Aly6 gradually cleaves one saturated M unit or continuously cleaves unsaturated ∆ units from the *nr* end of substrate chains. Furthermore, the 2-AB-M5 substrate chain was firstly digested into hypothetical ∆ and 2-AB-UM4 or M2 and 2-AB-UM3 product chains by endolytic enzymes Pae-rAlgL or Avi-rAlgL (Figure 4D,E) and finally into fluorescently detectable products of 2-AB-UM3 and 2-AB-UM2. Additionally, for the 2-AB-M4 substrate, ∆ and 2-AB-UM3 or M2 and 2-AB-UM2 chains were possible products, among which 2-AB-UM3 was the final main detectable product (Figure S6). These results indicated that Pae-AlgL or Avi-AlgL feature unstrict (variable) endo-type modes.

**Figure 5:**
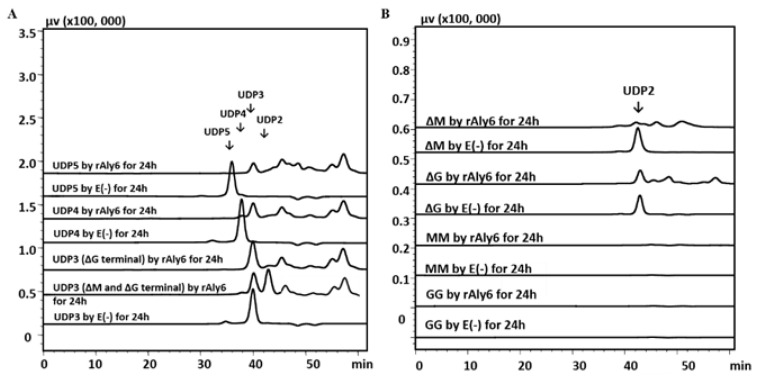
Gel filtration HPLC analyses of oligosaccharide degradation by rAly6. (**A**) intermediate unsaturated oligosaccharide products of rAly6 (UDP3-UDP5 fractions); (**B**) disaccharides; E (−), control treated with the inactivated enzyme of rAly6.

The substrates of the ΔG-terminal UDP5 and UDP4 fractions were fully digested into UDP3 and UDP2 products by rAly6; however, ΔG-terminal UDP3 fractions could only be partially degraded, in molar amounts of ~5%, while the ΔM/ΔG-terminal UDP3 substrate was digested at ~60% (Figure 5A). Furthermore, ΔM (the disaccharide) was the smallest tested unsaturated oligosaccharide substrate of rAly6 (Figure 5B), and ΔM was more easily degraded than ΔG (Table 2). Thus, Aly6 was an M-preferred exo-type alginate lyase, preferring to degrade oligosaccharide substrates larger than trisaccharide and could digest ΔM-terminal rather than ΔG-terminal substrates.

### 2.5. Catalytic Mechanism of Aly6

**Figure 6:**
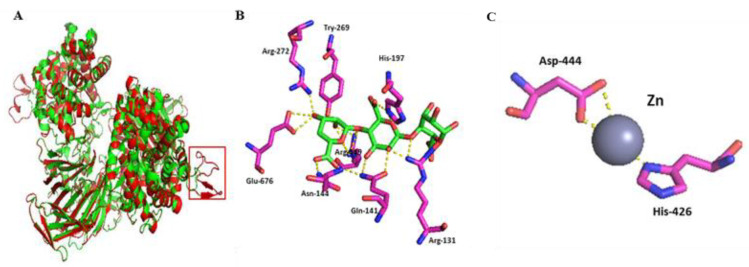
Homology-based protein structure modeling and molecule stocking of Aly6. (**A**) Structural comparison of Aly6 (red) and Alg17c (green) and the extra peptide segment (red box); (**B**) oligosaccharide ligands within 4 Å; (**C**) metal ions (Zn) within 4 Å. The ligands BEM-MAV-LGU and Zn of the Y258A (PDB: 4OJZ) mutant of Alg17c were used to predict active site residues of Aly6. BEM-MAV-LGU, unsaturated mannuronic acid-mannuronic acid-guluronic acid.

In the homology-based protein structure modelling tests, Aly6 was composed of α6/α6 barrels and antiparallel β-chains as a sheet (Figure S7A) with a crack-like catalytic cavity, using Alg17c (PDB: 4NEI) as template (Figure S7B), which was significantly different from the barrel or jelly roll folds used by other PL family members. After gene truncation of the peptide Gly^224^~Ala^248^ (Figure 6A), there was little enzyme activity change (Table 3) in the protein mutants, indicating that Gly^224^~Ala^248^ is not a core essential structure of the protein Aly6. However, the H197A and W200A protein mutants showed individual activities against alginate of ~46 ± 4.6 and ~44 ± 3.1 U/mg (Table 3), whereas H426A, Y269A, and N144A were approximately completely inactivated (Table 3), indicating that HNH^197^STW^200^ is a catalytic, half-conserved, and essential motif of Aly6 (Figure S1). Therefore, Aly6 represents a novel member of the PL17 subfamily.

## 3. Discussion

In this study, to elucidate and compare the core values of alginate lyases with M-preference in alginate degradation, direct oligosaccharide preparation, and associated enzymatic mechanisms, and to benefit novel tool-like enzyme exploration, we report comparative studies of Aly6 (AlgL-like module and Hep_II_III-like module) as a novel M-preferred exolytic enzyme of strain MY04 (Figure 1C) and Pae-AlgL and Avi-AlgL (AlgL catalytic module) as two typical M-specific endolytic enzymes (Figure 1A,B). There were very low identities among the proteins, Aly6, Pae-AlgL and Avi-AlgL. Furthermore, this study demonstrated that the AlgL-like module and the Hep_II_III-like module in Aly6 both played indispensable roles in determining enzyme characteristics. We also found that the active, half-conserved, and essential motif of the exolytic enzyme Aly6 is HNHSTW, which is different from those in the PL5 and PL17 families. Interestingly, our data also demonstrated that AlgL and AlgL-like modules played essential roles in similar M-preferring substrate selectivity, although the Aly6 and PL5 proteins and other PL17 family members differed greatly in their sequence properties (Figure 1D, Table S2).

The first exo-type alginate lyase characterized to produce ΔG-terminal oligosaccharide chains as intermediate products was Aly6 (Figure 2C), whose oligosaccharide-yielding properties differed significantly from the enzymatic preparation properties reported for the endo-type enzymes, e.g., Aly5 [29], Aly1 [30], and Aly2 [31] of *Flammeovirga* sp. strain MY04. Interestingly, we reported that the M-specific endolytic lyases, Pae-AlgL and Avi-AlgL, featured a succession rule in the final alginate digestions, changing from the ∆M- to ∆G-terminus, while the product size increased (Figure 2A,B). To our knowledge, this is the first report on the oligosaccharide-yielding property of an M-specific endolytic lyase. Furthermore, our tests confirmed that Aly6 gradually removed one saturated M unit or two continuous unsaturated Δ units from the *nr* end of a substrate chain using monosaccharide exolytic behavior, which was similar to the versatile monosaccharide-producing G-preferred endolytic enzyme Aly2 of strain MY04 (Figure 4A–C) [31]. However, two endo-type enzymes, Pae-AlgL and Avi-AlgL, also showed variable substrate degradation patterns affected by the substrate terminus types, DPs, and M/G ratios (Figure 3C,D and Figure 4D,E); furthermore, they eventually yielded succession-ruled oligosaccharide products (Figure 2A,B).

In most cases, compared with the endolytic enzymes, the exolytic enzymes showed lower enzymatic activity and stability towards polysaccharides, possibly because the polysaccharides were too large to be efficiently bound to by exolytic enzymes. Our results demonstrated the action modes and underlying catalytic mechanisms of a novel M-preferred monosaccharide-yielding exolytic enzyme, Aly6, with ~726 ± 2.2 U/mg activity against alginate, and two typical endolytic enzymes, Pae-AlgL (~2685 ± 3.6 U/mg) and Avi-AlgL (~4219 ± 1.8 U/mg) (Table 1). This study provided new insights into three high-activity alginate lyases in terms of their different core values in the direct preparation of oligosaccharide fractions with designated components and molecular sizes. The study could benefit the commercial production and enzymatic application of M-preferring alginate lyase and make a very important contribution to novel resource discoveries, oligosaccharide preparations, and sequence determinations.

## 4. Materials and Methods

### 4.1. Bacterial Strains, Carbohydrates, and Growth Conditions

The *Escherichia coli* strain cells, DH5α or BL21(DE3), were cultured at 37 °C for gene cloning and at 16 °C for protein expression in Luria-Bertani (LB) broth supplemented with ampicillin, at a final concentration of 100 μg/mL, or with kanamycin, at 50 μg/mL. When necessary, *Flammeovirga* sp. strain MY04 (CGMCC No. 2777) was cultured at 30 °C in a medium (pH 7.0) comprising (*w*/*v*) 0.40% tryptone, 0.25% yeast extract, and 3.0% NaCl [47]. Solid medium plates were prepared by supplementing additional agar powder (1.5%, *w*/*v*).

The agarose, alginate (CSA: 9005-38-3. Lot# SLBQ3067V. viscosity: ≥ 2000 cP, 2% (25 °C)), chondroitin, chondroitin sulfates (A, C, D, and E types), hyaluronan (HA), heparin, heparin sulfate (HS), pectin, and xanthan were purchased from Sigma-Aldrich Co., Ltd., Boston, MA, USA. The poly-G blocks, poly-M blocks, and standard size-defined G-enriched or M-enriched saturated sugar chains (ranging from disaccharide to heptasaccharide in size, with >95% promised purities) were purchased from Biozhi Biotech Co., Ltd., Qingdao, China. Various size-defined unsaturated oligosaccharide fractions were prepared using the alginate lyases rAly5 and rAly1 as described in previous studies [29,30] or using recombinant alginate lyases rAly6, Pae-rAlgL, and Avi-rAlgL in this study.

### 4.2. Gene and Protein Sequences

The DNA sequence of ORF2549 from the genome of *Flammeovirga* sp. strain MY04 was translated into the amino acid sequence of the Aly6 protein, and the GC content (G+C%) was calculated using BioEdit version 7.2.5 (http://www.mbio.ncsu.edu/BioEdit/bioedit.html, access on 1 October 2021). The signal peptides were analyzed using SignalP server 4.1 (http://www.cbs.dtu.dk/services/, access on 1 December 2021). The molecular weights and the pI of the protein were estimated using the peptide mass tool on the ExPASy server of the Swiss Institute of Bioinformatics (http://swissmodel.expasy.org/, access on 1 December 2021). Online similarity searches on the Aly6 sequence were performed using the BLAST algorithm on the National Center for Biotechnology Information server (http://www.ncbi.nlm.nih.gov, access on 1 December 2021). The protein modules and functional domains were identified using the Simple Modular Architecture Research Tool (https://en.wikipedia.org/wiki/Simple_Modular_Architecture_Research_Tool, access on 1 December 2021), the Pfam database (http://pfam.xfam.org, access on 1 December 2021), and the CAZy database (http://www.cazy.org, access on 1 December 2021). Multiple sequence alignments and phylogenetic analyses were performed with each protein sequence using MEGA version 7.2.5 (Mega Limited, Auckland, New Zealand). Two wild-type DNA sequences that encode periplasmic AlgL proteins of *Pseudomonas. aeruginosa* (GenBank: Nucleotide U27829.1/Protein AAA91127.1) and *Azotobacter vinelandii* (GenBank: Nucleotide AF027499/Protein AAC04567.1) were analyzed as described above.

### 4.3. Construction of Expression Vectors

The genomic DNA of the *Flammeovirga* sp. strain MY04 was prepared and purified using the FastPure bacterial DNA isolation mini kit (Vazyme Biotech Co., Ltd., Nanjing, China). To express the whole Aly6 protein, the full-length gene was amplified from the genomic DNA using the high-fidelity Vazyme™ LAmp DNA poly-Merase (Vazyme Biotech Co., Ltd., Nanjing, China) and the primers TF-Aly6-F and TF-Aly6-R (Table S1). The PCR product was finally enzyme-digested (Nde I and Xba I) and cloned into the expression vector pET-30a (+) or pColdTF (Table S1), producing the recombinant plasmids pET30-Aly6 and pCTF-Aly6 to yield the full-length protein rAly6 and the TF-factor-fused protein rTF-Aly6, respectively. To express the soluble proteins of the putative *N*-terminal module (AlgL module) and the *C*-terminal module (heparinase II or III module) of Aly6, i.e., the recombinant proteins of rTF-Aly6-Lmodule and rTF-Aly6-HPmodule, the primers (Table S1) were designed and used in the construction of recombinant plasmids pCTF-Aly6-Lmodule and pCTF-Aly6-HPmodule, respectively.

The wild-type Pae-AlgL and Avi-AlgL genes were optimized for G+C contents and nucleotide codons, artificially synthesized, and finally directly cloned into the plasmid vector pET-30a (+) between Nde I and Xho I restriction sites, thus forming recombinant plasmids pET30-Pae-AlgL and pET30-Avi-AlgL to produce recombinant proteins of Pae-rAlgL and Avi-rAlgL, respectively.

### 4.4. Heterologous Expression and Purification of Recombinant Proteins

Each recombinant plasmid was transformed into *E. coli* BL21(DE3) cells. To initiate protein expression, the LB broth was supplemented with isopropyl 1-thio-β-d-galactoside (IPTG) to a final concentration of 0.05 mM when the A_600_ reached ~0.8. After continual 24 h cultivation at 16 °C, the cells were harvested by centrifugation at 8000× *g* for 10 min, washed twice using an ice-cold buffer A (50 mM Tris, 150 mM NaCl, pH 8.0), resuspended in buffer A, and disrupted by sonication (60 repetitions, 5 s). After centrifugation at 15,000× *g* for 30 min, the supernatant containing each soluble protein was loaded onto a buffer A-equilibrated Ni-nitrilotriacetic acid agarose (Ni-NTA) column (TaKaRa, Dalian, China). Subsequently, each column was eluted using buffer A containing imidazole at increasing concentrations, e.g., 0, 10, 50 and 250 mM. The purified protein fractions were dialyzed against buffer B (50 mM Tris, 50 mM NaCl, 5% glycerol (*v*/*v*), pH 8.0).

Fractionated protein samples were analyzed using 13.2% (*w*/*v*) polyacrylamide gel SDS-PAGE [48]. Protein concentrations were individually determined by the Folin-–Lowry method using Folin and Ciocalteu’s phenol reagent (Sigma-Aldrich, USA) with bovine serum albumin as the standard. The protein bands were converted into peak area integrals to determine purifications by Adobe^®^ Photoshop^®^ CS5 software (Adobe Systems Inc., San Jose, CA, USA).

### 4.5. Enzyme Activity Assays

To determine the substrate preference of each purified recombinant protein, various polysaccharides and oligosaccharides were individually dissolved in deionized water to prepare stock solutions (3 mg/mL). Each stock solution (100 μL) was mixed with 30 μL of appropriately diluted enzyme preparation, 100 μL of 150 mM NaAc-HAc buffer (pH 6.0), and 70 μL of water. Each reaction was performed at 40 °C for 12 h. The enzyme-treated samples were heated in boiling water for 10 min and subsequently ice-cooled. After centrifugation at 15,000× *g* for 15 min, the supernatant was collected and analyzed by measuring the absorbance of DNS-reducing sugars at 540 nm. One unit was defined as the amount of enzyme required to produce 1 μmol reducing sugars per minute, and the samples (1.0 mg/mL) were further analyzed by gel filtration on a Superdex^TM^ peptide 10/300 GL column or a Superdex^TM^ 30 Increase 10/300 GL column (GE Healthcare, Chicago, IL, USA). The absorbance was monitored at 235 nm using a UV detector. The mobile phase was 0.2 M NH_4_HCO_3_, and the flow rate was 0.4 mL/min. Online monitoring and data analysis were performed using LCsolution version 1.25 software (Shimadzu Corporation, Kyoto, Japan).

### 4.6. Biochemical Characterization of Recombinant Proteins

To determine the optimal temperature for the alginate lyase activities, various polysaccharides, alginate, poly-G blocks, and poly-M blocks, were individually reacted with protein preparations. The enzymatic reactions were performed in a 50 mM NaAc-HAc buffer (pH 6.0), at temperatures ranging from 0 °C to 80 °C, for 90 min. After the optimal temperature was determined, the effects of the pH values on the enzyme activities were tested in different buffers, including a 50 mM NaAc-HAc buffer (pH 5.0 and 6.0), a 50 mM NaH_2_PO4-Na_2_HPO_4_ buffer (pH 6.0, 7.0, and 8.0), and a 50 mM Tris-HCl buffer (pH 7.0, 8.0, 9.0 and 10), each with a total volume of 300 μL. The thermostability was evaluated by measuring the residual enzyme activity of each enzyme preparation after incubation for 0 h to 24 h at 0 to 80 °C. The effects of the pH values on the enzyme stabilities were determined by measuring the residual activity of each enzyme after incubation at 4 °C at varying pH values (5.0–10) for 2 h. The effects of the metal ions and chelating agents on the alginate lyase activities were examined by determining the activity of each enzyme in the presence of 1 mM metal ions or 10 mM chelating agents. The effects of NaCl on the alginate lyase activities were examined by determining the activity of each enzyme at different concentrations (from 0.0 M to 1.0 M). All the reactions were performed in triplicate. After each treatment, the enzyme activity was estimated as described in Section 4.5.

### 4.7. Comparison of Polysaccharide-Degrading Patterns

To compare the polysaccharide degradation patterns of recombinant proteins, the alginate (1.0 mg/mL) digestion by each enzyme (1.0 U/mL) at 40 °C was traced over 72 h. Similar experiments were performed at alginate concentrations ranging from 1.0 mg/mL to 10 mg/mL. The aliquots from the digestions were removed for time-course analysis. To determine the molar ratio of each oligosaccharide fraction in the products, the samples (1.0 mg/mL) were analyzed as described in Section 4.5.

To determine the oligosaccharide compositions of the final alginate degradation products, 100 mg of alginate (1.0 mg/mL) was initially digested by an excess of each recombinant enzyme (10 U/mL) under their optimal conditions for more than 72 h. To further obtain each size-defined unsaturated oligosaccharide product fraction, the final alginate degradation products by Pae-rAlgL and Avi-rAlgL or the intermediate alginate digests by rAly6 or rTF-Aly6 were individually gel-filtered through a Superdex^TM^ 30 Increase 10/300 GL column using the same protocol as described in Section 4.5. Each fraction was collected and freeze-dried repeatedly to remove NH_4_HCO_3_ for further analysis. The molecular mass of each oligosaccharide fraction was determined by matrix-assisted laser desorption/ionization time-of-flight mass spectrometry (AXIMA-CFR plus, Shimadzu, Japan). For ^1^H-NMR spectroscopy, each purified oligosaccharide fraction (~2 mg) was dissolved in 0.3 mL of D_2_O in 5-mm NMR tubes. The spectra were recorded on a JNM-ECP600 (JEOL, Tokyo, Japan) apparatus set at 600 MHz using TMS as the internal standard.

### 4.8. Comparison of Oligosaccharide Degradation Patterns

To determine the smallest substrate of each recombinant enzyme, unsaturated oligosaccharides with different DPs, e.g., UDP2, UDP3, UDP4, UDP5, UDP6, and UDP7 fractions, were first purified from the final alginate digests by various endolytic alginate lyases, e.g., rAly1 (bifunctional but G-preferred) [30] and rAly5 (G-specific) [29] of strain MY04. In addition, unsaturated oligosaccharide fractions were also purified from alginate that had been completely digested by recombinant enzymes Pae-rAlgL and Avi-rAlgL or from alginate that had been partially digested by recombinant enzymes rAly6 or rTF-Aly6 as described in Section 4.7. Next, the obtained oligosaccharide fractions were used as testing substrates of each recombinant enzyme preparation. To further determine the substrate preference and the oligosaccharide degradation pattern of each enzyme, standard saturated size-defined M-enriched and G-enriched sugar chains were individually reacted with recombinant enzyme preparations. The tested natural substrates and their enzymatic products (20 µg each) were subjected to the gel filtration HPLC assay described in Section 4.5.

To fully compare the enzymatic degradation pattern of each recombinant enzyme preparation, various size-defined saturated and unsaturated oligosaccharide fractions were fluorescently labeled at their reducing (*r*) ends using excess 2-aminobenzamide (2-AB, Sigma-Aldrich, Boston, MA, USA) [42]. Artificially labeled products (~1 µg each) were purified by gel-filtration HPLC and further reacted with each enzyme (5 U) in a total volume of 1 mL using the protocol described in Section 4.7. The above artificial substrates and their final enzymatic products (50 ng each) were subjected to gel filtration HPLC assay as described in Section 4.5 and monitored with a fluorescence detector. The excitation and emission wavelengths were set at 330 nm and 420 nm, respectively.

### 4.9. Analysis of the Catalytic Mechanism of the Recombinant Proteins

SWISS-MODEL software (http://swissmodel.expasy.org/, accessed on 1 December 2021) was used for homology-based protein structure modeling. Next, the three-dimensional structure of Aly6 was analyzed, and the active site residues of the protein were predicted by PyMOL (http://pymol.org/, accessed on 1 December 2021). Mutant primers (Table S1) were designed and applied for reverse PCR using the recombinant plasmid pET30-Aly6 as a template, and mutant recombinant plasmids were sequenced and named pET30-Aly6-N144A, pET30-Aly6-H197A, pET30-Aly6-W200A, pET30-Aly6-Y269A, pET30-Aly6-H426A, and pET30-Aly6-T-G^224^-A^248^. The methods of plasmid construction, expression, and enzyme activity analysis are described above.

## Figures and Tables

**Table 1:** Activities of alginate lyases (U/mg).

Enzymes	Alginate	Poly-M	Poly-G
rAly6	726 ± 2.2	525 ± 3.5	196 ± 2.9
rTF-Aly6	692 ± 2.7	547.9 ± 3.3	183 ± 3.1
rTF-Aly6-Lmodule	34.6 ± 5.1	25.1 ± 4.2	5 ± 4.2
rTF-Aly6-HPmodule	48 ± 4.9	19.5 ± 6.4	8 ± 6.1
Pae-rAlgL	2685 ± 3.6	5704 ± 4.5	144 ± 3.3
Avi-rAlgL	4219 ± 1.8	8085 ± 5.9	98 ± 5.2

**Table 2:** Oligosaccharides degraded by rAly6.

Test Substrate	Product (s)	Degradation Ratio
ΔG	Δ	5%
ΔM	Δ	90%
GG	---	---
MM	---	---
ΔGX/ΔMX	UDP2	60%
ΔGX	UDP2	5%
ΔGXX	UDP3 and UDP2	95%
ΔGXXX	UDP3 and UDP2	98%

Δ is an unsaturated sugar unit derived from either the saturaed M or G monosaccharides.

**Table 3:** Aly6 mutant activity analyses.

Enzymes	Mutants	Activity (U/mg)
Metal ions	H426A	0
Extra peptide	G^224^-A^248^	367 ± 2.2
NNHSYW^200^	W200A	144 ± 3.1
Active site residues	H197A	46 ± 4.6
	Y231A	566 ± 1.9
	Y269A	9 ± 5.4
	N144A	16 ± 4.8

## Supplementary Materials

The following are available online at https://www.mdpi.com/article/10.3390/md19120706/s1. Figure S1. Sequence alignment of Aly6 and characterized PL17 alginate lyases. Sequence alignment was performed using DNAMAN software via multiple sequence alignment. Figure S2. Purification of recombinant proteins from *E. coli* cells by Ni^2+^ chelation chromatography. A, rTF-Aly6. B, rAly6. C, rTF-Aly6-Lmodule. D, rTF-Aly6-HPmodule. E, Pae-rAlgL. F, Avi-rAlgL. Lane M, PierceTM unstained protein molecular weight marker, product# 26610 (Thermo Scientific); lane 1, cell lysate of induced *E. coli* cells harboring the control plasmid pET-30a (+) or pColdTF; lane 2, cell lysate of induced *E. coli* cells containing each recombinant plasmid; lane 3, supernatant of induced cell lysates; lane 4, purified proteins. Figure S3. Biochemical characteristics of the enzyme rAly6. A, Optimal temperature.). B, Effects of pH and pH stability. C, Thermostability of rAly6. D, Effects of NaCl. E, Effects of various compounds on enzyme activity. Activities are shown as the activities relative to that of untreated enzyme. Error bars represent the mean values of triplicates ± SDs. Figure S4. Biochemical characteristics of Pae-rAlgL and Avi-rAlgL. A, Effects of various compounds on the enzyme activity of Pae-rAlgL. B, Effects of various compounds on the enzyme activity of Avi-rAlgL. C, Effects of NaCl on the enzyme activity of Pae-rAlgL. D, Effects of NaCl on the enzyme activity of Avi-rAlgL. Activities are shown as the activities relative to that of untreated enzyme. Error bars represent the mean values of triplicates ± SDs. Figure S5. Degradation pattern analyses of alginate and associated unsaturated oligosaccharide substrates. A, Time-course of alginate degraded by Pae-rAlgL. B, Time-course of alginate degraded by Avi-rAlgL. C, Time-course of alginate degraded by rAly6. D, Analyses of the degradation of unsaturated oligosaccharides by Pae-rAlgL and Avi-rAlgL. E (-), control treated with the inactivated enzyme. Figure S6. Fluorescent analyses of the degradation patterns of Pae-rAlgL and Avi-rAlgL. A, Time-course of 2-AB-M4 degradation by Pae-rAlgL. B, Time course of 2-AB-M4 degradation by Avi-rAlgL. The resulting products were analyzed by gel filtration HPLC using a fluorescent detector with an excitation wavelength of 330 nm and an emission wavelength of 420 nm. E (−), control treated with inactivated enzyme. Figure S7. Homology modeling of the Aly6 protein structure. A, Homologous modeling of the three-dimensional structure of Aly6 based on Alg17c (PBD: 4NEI). B, Cartoon model. Red, α-helixes; yellow, β-strands; green, loops. Table S1. Bacterial strains, plasmids, and primers used in the present study. Table S2. The identities of Aly6 and characterized alginate lyases.
