# Supplementary material for: Comparison of Biochemical Characteristics, Action Models, and Enzymatic Mechanisms of a Novel Exolytic and Two Endolytic Lyases with Mannuronate Preference
Source: Mar Drugs. 2021 Dec 14;19(12):706. doi: 10.3390/md19120706 (PMC8705907; doi:10.3390/md19120706)
Supplement: Supplementary file 1 [file marinedrugs-19-00706-s001.zip › marinedrugs-1513553-supplementary.pdf]

Supplementary data

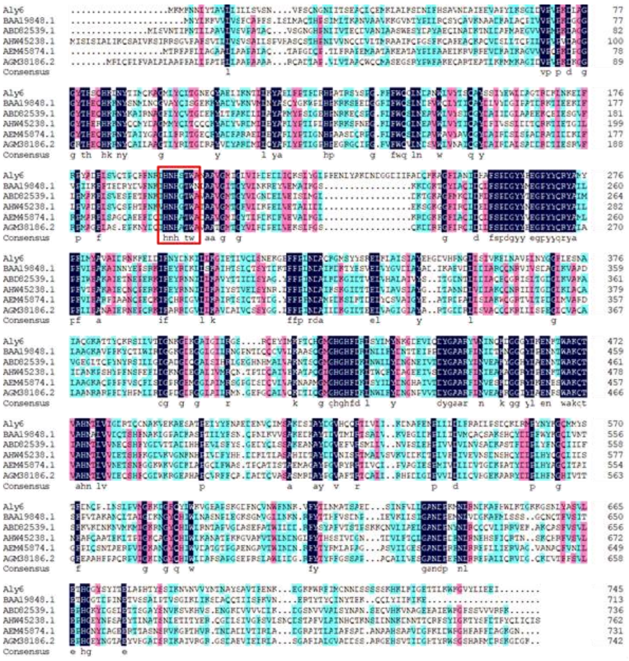

Figure S1. Sequence alignment of Aly6 and characterized PL17 alginate lyases. Sequence alignment was performed using DNAMAN software via multiple sequence alignment.

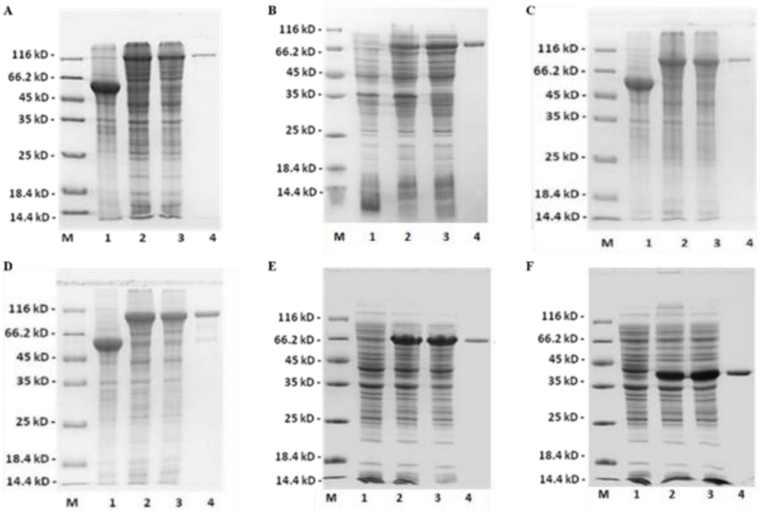

Figure S2. Purification of recombinant proteins from *E. coli* cells by Ni^{2+} chelation chromatography. A, rTF-Aly6. B, rAly6. C, rTF-Aly6-Lmodule. D, rTF-Aly6-HPmodule. E, Pae-rAlgL. F, Avi-rAlgL. Lane M, PierceTM unstained protein molecular weight marker, product# 26610 (Thermo Scientific); lane 1, cell lysate of induced *E. coli* cells harboring the control plasmid pET-30a (+) or pColdTF; lane 2, cell lysate of induced *E. coli* cells containing each recombinant plasmid; lane 3, supernatant of induced cell lysates; lane 4, purified proteins.

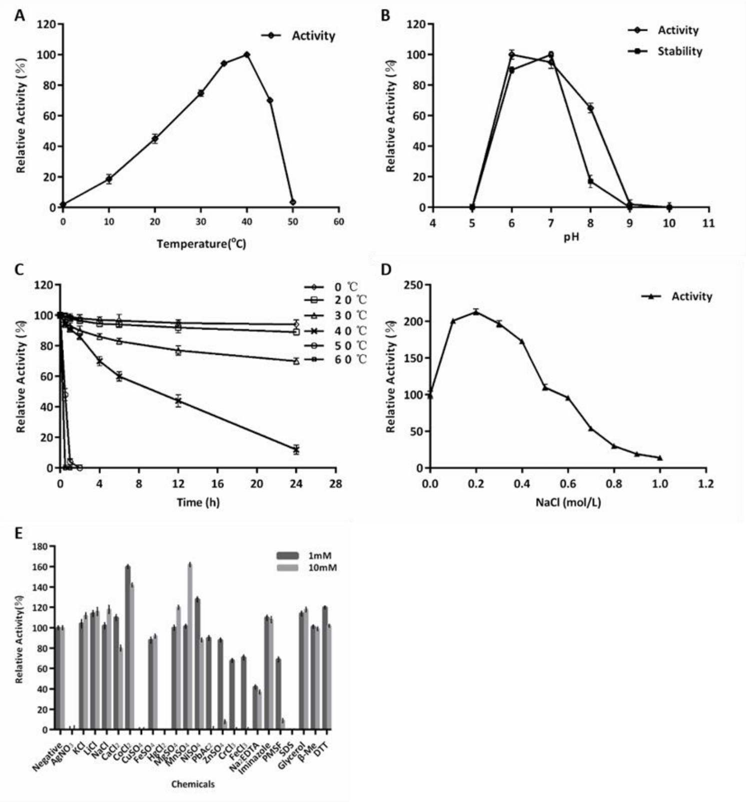

Figure S3. Biochemical characteristics of the enzyme rAly6. A, Optimal temperature.). B, Effects of pH and pH stability. C, Thermostability of rAly6. D, Effects of NaCl. E, Effects of various compounds on enzyme activity. Activities are shown as the activities relative to that of untreated enzyme. Error bars represent the mean values of triplicates ± SDs.

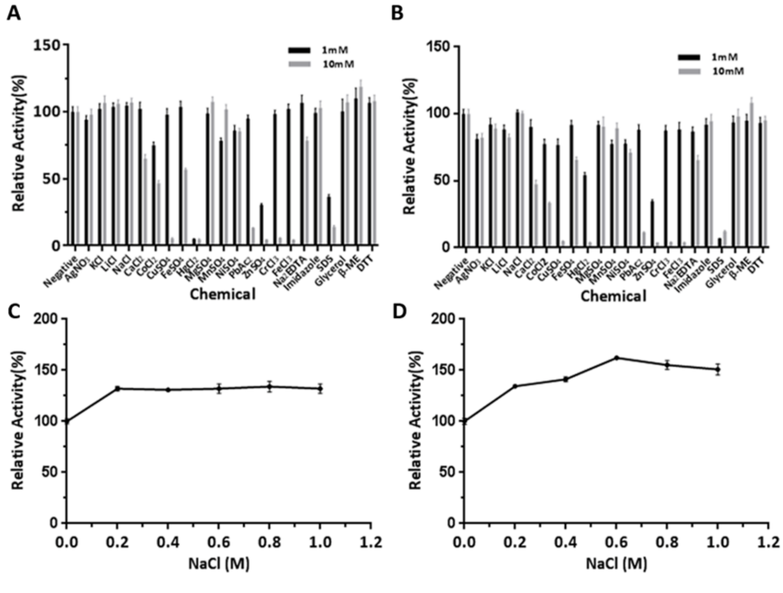

Figure S4. Biochemical characteristics of Pae-rAlgL and Avi-rAlgL. A, Effects of various compounds on the enzyme activity of Pae-rAlgL. B, Effects of various compounds on the enzyme activity of Avi-rAlgL. C, Effects of NaCl on the enzyme activity of Pae-rAlgL. D, Effects of NaCl on the enzyme activity of Avi-rAlgL. Activities are shown as the activities relative to that of untreated enzyme. Error bars represent the mean values of triplicates ± SDs.

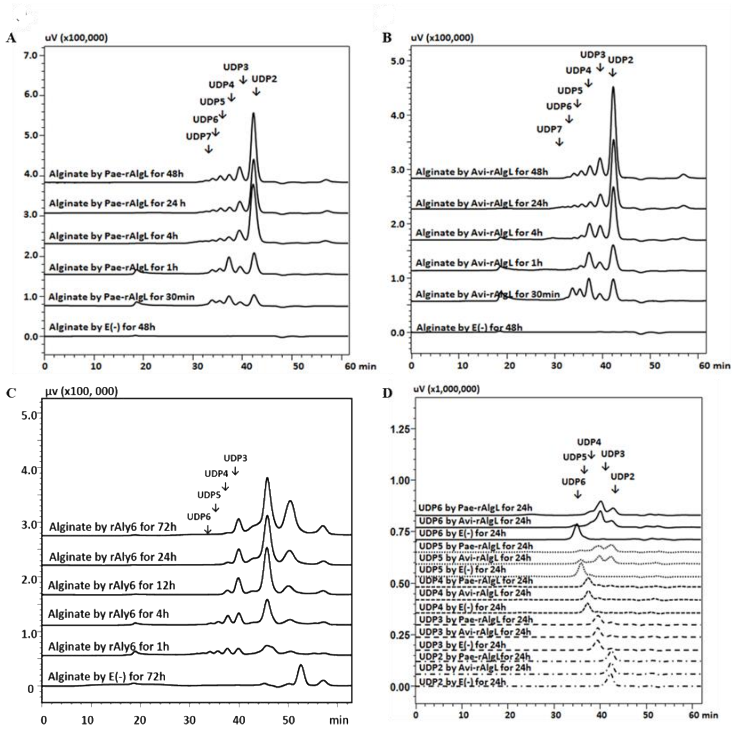

Figure S5. Degradation pattern analyses of alginate and associated unsaturated oligosaccharide substrates. A, Time course of alginate degraded by Pae-rAlgL. B, Time-course of alginate degraded by Avi-rAlgL. C, Time-course of alginate degraded by rAly6. D, Analyses of the degradation of unsaturated oligosaccharides by Pae-rAlgL and Avi-rAlgL. E (-), control treated with the inactivated enzyme.

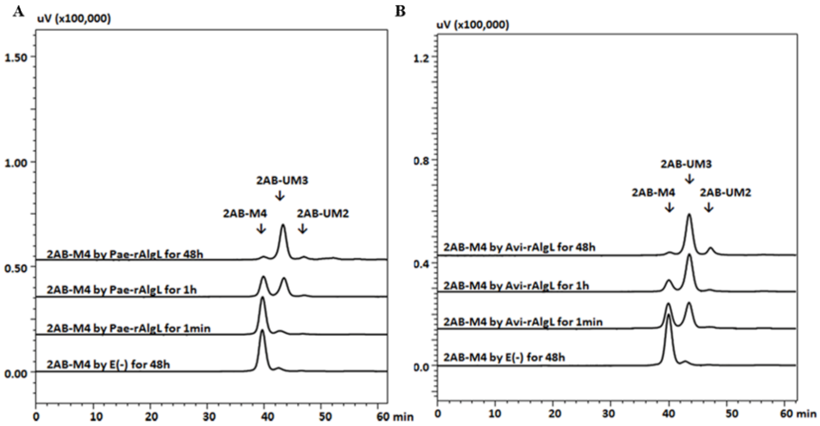

Figure S6. Fluorescent analyses of the degradation patterns of Pae-rAlgL and Avi-rAlgL. A, Time course of 2-AB-M4 degradation by Pae-rAlgL. B, Time course of 2-AB-M4 degradation by Avi-rAlgL. The resulting products were analysed by gel filtration HPLC using a fluorescent detector with an excitation wavelength of 330 nm and an emission wavelength of 420 nm. E (-), control treated with inactivated enzyme.

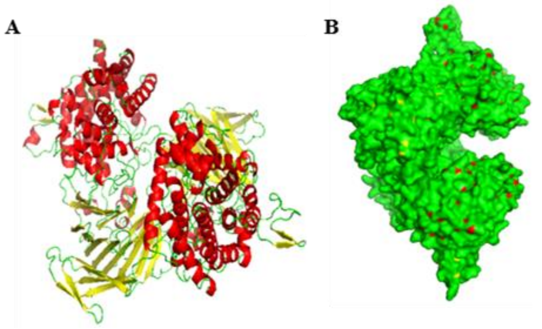

Figure S7. Homology modeling of the Aly6 protein structure. A, Homologous modeling of the three-dimensional structure of Aly6 based on Alg17c (PBD: 4NEI). B, Cartoon model. Red, α-helixes; yellow, β-strands; green, loops.

Table S1. Bacterial strains, plasmids, and primers used in the present study.

Strains and plasmids	Description	Source
Flammeovirga sp. strain MY04	A polysaccharide-degrading marine bacterium	CGMCC No. 2777
E. coli BL21(DE3)	F- ompT hsdS (rB-, mB-) gal dcm (DE3)	Vazyme
E. coli DH5α	lacZΔM15, endA1, RecA1	Vazyme
pET-30a (+)	N-His, N-S, N-Thrombin, N-EK, C-His	TaKaRa
pColdTF	N-His, N-Trigger factor, N-HRV 3C, N-Thrombin, N-Factor Xa	TaKaRa
Nde I	5'-CATATG-3'	TaKaRa
Xho I	5'-CTCGAG-3'	TaKaRa
Xba I	5'-TCTAGA-3'	TaKaRa
pCTF-Aly6<br>pCTF-Aly6-HPmodule<br>pCTF-Aly6-Lmodule	Carrying an amplified Nde I-Xba I fragment encoding the recombinant protein fused with a His6 tag at the C terminus	This study
pET30-Aly6<br>pET30-Pae-AlgL<br>pET30-Avi-AlgL	Carrying an amplified Nde I- Xho I fragment encoding the recombinant protein fused with a His6 tag at the C terminus	This study
TF-*Aly6*-F	5'-ggCATATGCAGAACGGAAATTTAATTACTTCCG-3'	Sangon
TF-*Aly6*-R	5'-gcTCTAGAAATTTCTTCCAATAGGTAAACCCC-3'	Sangon
TF-*Aly6*-Lm-F	5'-ggCATATGCAGAACGGAAATTTAATTACTTCCGAG-3'	Sangon
TF-*Aly6*-Lm-R	5'-gcTCTAGACAAACTTCTTTTTTGGTAAGTCGTTGC-3'	Sangon
TF-*Aly6*-HPm-F	5'-ggCATATGTACTATTCAAGAGAATTGAAACTAGCC-3'	Sangon
TF-*Aly6*-HPm-R	5'-gcTCTAGAAATTTCTTCCAATAGGTAAACCCC-3'	Sangon
Aly6-N144A-F	5'-TTTAgcaGATGCCAATTGGTTGGTTTATAC-3'	Sangon
Aly6-N144A-R	5'-AATTGGCATCtgcTAAACACTGCCAGAAAAATTTAC-3'	Sangon
Aly6-H197A-F	5'-CTGCATAACgcaAGTACTTGGGCAAATGCAGC-3'	Sangon
Aly6-H197A-R	5'-GTACTtgcGTTATGCAGACGGTTAAAGAATTG-3'	Sangon
Aly6-W200A-F	5'-AGTACTgcaGCAAATGCAGCAGTAGGTATG-3'	Sangon
Aly6-W200A-R	5'-GCATTTGCtgcAGTACTATGGTTATGCAGACGG-3'	Sangon
Aly6-Y269A-F	5'-AGGTCCCgcaTACCAAAGATATGCGATGTATCC-3'	Sangon
Aly6-Y269A-R	5'-TTTGGTAtgcGGGACCTTCGAAATAATATCC-3'	Sangon
Aly6-H426A-F	5'-ACATGGTgcaTTCGACCGCCTTTCTTATTTG-3'	Sangon
Aly6-H426A-R	5'-GGTCGAAtgcACCATGTCCCATACCGTGTTG-3'	Sangon
Aly6-T-G^{244}-A^{248}-F	5'-AAATCTTTATACGGCTTCCTTGCCAACATCG-3'	Sangon
Aly6-T-G^{244}-A^{248}-R	5'-GAAGCCGTATAAAGATTTCTGAATTAAATCTTCATCG-3'	Sangon

Table S2. The identities of Aly6 and characterized alginate lyases.

Module	Protein	Identity
Hep_II_III-like	AlyPB2 of *Photobacterium* sp. strain FC615	38.71%
	Atu3025 of *Agrobacterium fabrum* strain C58	24.00%
	AlyFRB of *Falsirhodobacter* sp. strain Alg1	22.34%
AlgL-like	AlgA of *Pseudomonas* strain E03	23.27%
	AlgL-Pae of *Pseudomonas aeruginosa*	32.14%
	AlgL-Avi of *Azotobacter vinelandii*	35.71%
